# Assessing Suicide Reporting in Top Newspaper Social Media Accounts in China: Content Analysis Study

**DOI:** 10.2196/26654

**Published:** 2021-05-13

**Authors:** Kaisheng Lai, Dan Li, Huijuan Peng, Jingyuan Zhao, Lingnan He

**Affiliations:** 1 School of Journalism and Communication Jinan University Guangzhou China; 2 School of Communication and Design Sun Yat-Sen University Guangzhou China; 3 Department of Psychology Sun Yat-Sen University Guangzhou China; 4 Guangdong Key Laboratory for Big Data Analysis and Simulation of Public Opinion Guangzhou China

**Keywords:** suicide, suicide reporting, mainstream publishers, social media, WHO guidelines

## Abstract

**Background:**

Previous studies have shown that suicide reporting in mainstream media has a significant impact on suicidal behaviors (eg, irresponsible suicide reporting can trigger imitative suicide). Traditional mainstream media are increasingly using social media platforms to disseminate information on public-related topics, including health. However, there is little empirical research on how mainstream media portrays suicide on social media platforms and the quality of their coverage.

**Objective:**

This study aims to explore the characteristics and quality of suicide reporting by mainstream publishers via social media in China.

**Methods:**

Via the application programming interface of the social media accounts of the top 10 Chinese mainstream publishers (eg, People’s Daily and Beijing News), we obtained 2366 social media posts reporting suicide. This study conducted content analysis to demonstrate the characteristics and quality of the suicide reporting. According to the World Health Organization (WHO) guidelines, we assessed the quality of suicide reporting by indicators of harmful information and helpful information.

**Results:**

Chinese mainstream publishers most frequently reported on suicides stated to be associated with conflict on their social media (eg, 24.47% [446/1823] of family conflicts and 16.18% [295/1823] of emotional frustration). Compared with the suicides of youth (730/1446, 50.48%) and urban populations (1454/1588, 91.56%), social media underreported suicides in older adults (118/1446, 8.16%) and rural residents (134/1588, 8.44%). Harmful reporting practices were common (eg, 54.61% [1292/2366] of the reports contained suicide-related words in the headline and 49.54% [1172/2366] disclosed images of people who died by suicide). Helpful reporting practices were very limited (eg, 0.08% [2/2366] of reports provided direct information about support programs).

**Conclusions:**

The suicide reporting of mainstream publishers on social media in China broadly had low adherence to the WHO guidelines. Considering the tremendous information dissemination power of social media platforms, we suggest developing national suicide reporting guidelines that apply to social media. By effectively playing their separate roles, we believe that social media practitioners, health institutions, social organizations, and the general public can endeavor to promote responsible suicide reporting in the Chinese social media environment.

## Introduction

Suicide is a public health problem of global concern. More than 800,000 suicide deaths occur every year, meaning that more than 2000 people die by suicide every day [1]. To deal with this problem, the World Health Organization (WHO) has proposed the need to highly prioritize suicide prevention on global public health and public policy issues [2]. Suicide is a multidimensional issue related to various factors that can be facilitated by psychological, biological, social, and environmental factors [3,4]. Mass media coverage is a significant agent in the social construction of reality in today’s world, which may affect people’s exposure to suicide behaviors, especially for vulnerable groups [5]. Accordingly, researchers have determined that the role of mass media in suicidal behavior warrants serious and focused attention [6].

Research on the negative effect of suicide reporting (commonly known as the Werther effect) in the academic field can be traced back to 1974. Philips [7] found that publishing suicide stories in British and American newspapers led to an immediate increase in the number of suicides in the region. Since then, studies have confirmed the imitation effect caused by media reporting [8,9] and further illustrated that this imitation effect can be aggravated when suicide is repeatedly reported [10] or depicted sensationally and graphically [11] or when the subject is a celebrity [12,13]. Subsequently, scholars have noted the potential effect of the media on suicide prevention. Empirical research conducted in Switzerland [14], Australia [15], and Hong Kong [16] has indicated that promoting education by introducing reporting guidelines to media practitioners could effectively improve the quality of suicide reporting. By conducting long-term experiments in Austria, researchers found that the application of media guidelines and media campaigns in the city of Vienna led to a reduction of more than 80% in the subway suicide rate in 6 months [17-19].

Given the effectiveness of media guidelines in suicide prevention, in 2008, WHO and the International Association for Suicide Prevention released professional criteria for suicide reporting for media practitioners [20]. Consequently, some scholars have begun to evaluate the extent to which suicide reporting followed these guidelines; these evaluation studies were concentrated in Western countries and showed that the implementation of the guidelines varied [21,22]. Recently, research and evaluation on suicide reporting in Asia has increased; generally, studies have found a low level of compliance with WHO guidelines [23-26]. When assessing the quality of suicide reporting in Indian newspapers, Armstrong et al [24] found a considerable amount of harmful information and minimal educational information. If we consider that Asia has the largest suicide numbers on the globe [27], however, we find that the amount of empirical evidence we have on the quality of suicide reporting in the continent is quite limited.

Overall, the literature on the evaluation of suicide reporting has focused on traditional media, especially newspapers; with the emergence of Web 2.0 technology, however, social media is becoming an indispensable part of public information dissemination [28]. In the health field, people increasingly rely on social media for health communication, and social media plays a crucial role in the construction of general cognition, attitude, and behavior in the field [29,30]. Thus, many media outlets have allocated their resources toward social media platforms [31], and these accounts have shown tremendous influence. The Twitter account of the New York Times has nearly 500 million followers; similarly, People’s Daily has more than 100 million followers on Sina Weibo. Language style, staff structure, and frequency of posting of mainstream media on social media platforms differs from traditional media platforms [32-34], and research has shown that mainstream media’s language style on social media is more vivid, concise, and easy to understand [34]. Do the differences between traditional and social media platforms affect mainstream media’s reporting preferences or content quality? Based on this question, investigating how the mainstream media reports on public issues via social media has recently become a challenging research topic.

An empirical study found that American mainstream outlets showed preferential interest in disseminating information related to psychiatric disorders on Twitter, and media attention to different mental health disorders determines followers’ retweet responses [35]. Regarding suicide, a recent study evaluated news publishers’ reporting of suicide on Facebook and found that news articles often provided harmful elements to readers, whereas positive elements were relatively rare [36]. To the best of our knowledge, the study has been the only one to investigate news publishers’ suicide reporting on a social media platform, and it focused primarily on English-speaking countries. It remains unknown how mainstream media reports suicide via social media in different cultural contexts, especially in China.

Suicide remains a significant public health issue in China today. In 2019, nearly 140,000 people died by suicide in China, second only to India in the world [37]. Although a few scholars have assessed suicide reporting in China, these studies were limited to specific regions or focused on traditional media [23,25,38]. Using WHO guidelines, Fu et al [23] compared suicide reporting of newspapers in Hong Kong, Taiwan, and Guangzhou; the findings indicated that reports in the three regions were mostly inconsistent with WHO recommendations. Chu et al [25] evaluated suicide reporting in China’s most influential newspapers and internet-based media, and the results showed that compliance with the guidelines was very low for 4 of the 12 recommendations (eg, less than 5% of stories provided information on where a person could go for help). To the best of our knowledge, no nationwide research has investigated the compliance of suicide reporting published by Chinese mainstream publishers on social media platforms. Thus, the aim of this study is 2-fold and the research questions are as follows: What are the characteristics of suicide reporting of mainstream publishers via social media? What is the extent to which their suicide reporting follows WHO guidelines?

## Methods

### Newspapers Selected

We obtained the top 100 media list of comprehensive communication power from a 2018 national online survey [39]. Since the number of followers directly affects the visibility of reports, the number of followers on Weibo, one of the most popular platforms in China [40,41], can represent the publisher’s influence. We ranked publisher Weibo accounts according to the number of followers on November 1, 2019, and categorized them as national or local. We then selected the top 5 national (People’s Daily, China Daily, Global Times, Guangming Daily, and China Youth News) and local (Beijing News, Shanghai Morning Post, New Express, Yangtze Evening Post, and Southern Metropolis Daily) newspapers for further study. Due to access restrictions, we were not able to collect data from New Express and replaced it with Chutian Metropolis Daily, which ranked sixth in local newspapers. Each publisher had more than 10 million followers on their Weibo accounts.

### Data Extraction

**Figure 1:**
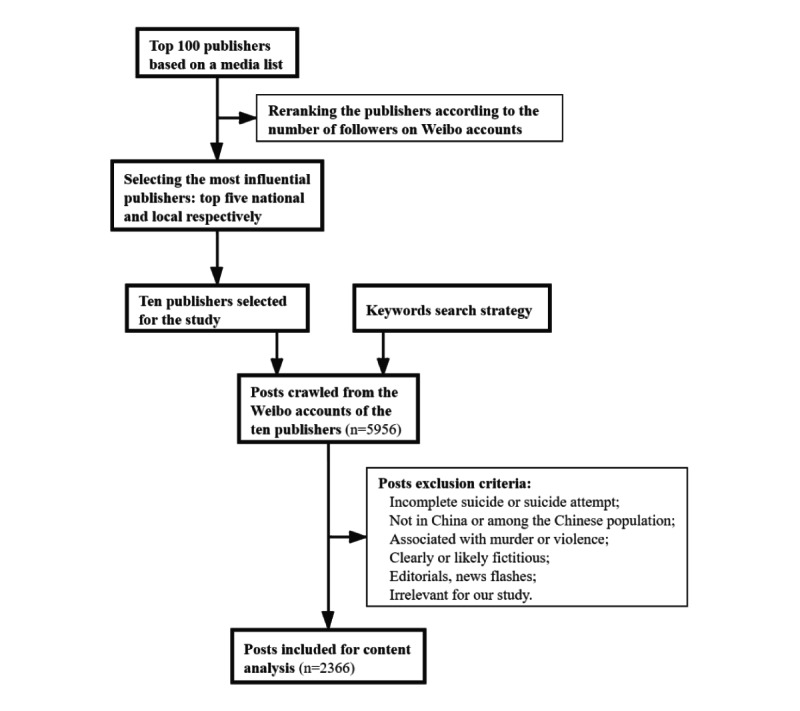
Flowchart of mainstream publishers selected and data extraction.

We used the open-source web data crawling tool Octopus to crawl the data by keyword search via the application programming interface of Weibo. Specifically, we designed a keyword search strategy that contained a core word (ie, “suicide”), a synonym (ie, “Zijin,” which means suicide in Chinese), and 8 hyponyms of common suicide methods in China (ie, “jumping off a building,” “jumping off a bridge,” “jumping into a river,” “drinking pesticides,” “taking drugs,” “wrist cutting,” “hanging,” and “charcoal burning”) [42]. Initially, we obtained 5956 posts published between August 11, 2011, and November 16, 2019, with at least one of the above keywords. These posts were then filtered based on the following criteria taken from prior research: event depicted in the post must have been a completed suicide or suicide attempt [24,25]; event must have occurred in China and among the Chinese population (ie, posts on foreigners who died by suicide in China and on Chinese people who died by suicide overseas were omitted) [23]; suicide events associated with murder or violence were omitted [25]; post must have been a narrative (ie, fictional, editorials, and single-sentence posts, called flashes, were omitted) [23]; and posts irrelevant for our study were omitted (eg, a post that stated “Staying up late equals to chronic suicide”). In total, we obtained 2366 posts (2363 original posts and 3 reposts) about suicide reporting (Figure 1).

### Content Analysis

We conducted content analysis based on prior evaluation research [21-26]. First, we extracted descriptive data to address the first research question that included the following 3 categories of suicide reporting characteristics: (1) text characteristics (ie, publisher name, publication time, and whether the post contained pictures or videos); (2) suicide event characteristics, including the type (ie, completed suicide or attempt), site (ie, urban or rural area), method (ie, jumping off a building, jumping off the bridge, jumping into the river, drinking pesticides, taking drugs, wrist cutting, hanging, charcoal burning, other ways, or multiple ways), and cause (ie, family conflict, emotional frustration, financial difficulty, interpersonal conflict, mental disorder, study or work pressure, to avoid responsibility, physical disease, loneliness or solitude, other causes, or multiple factors) [42]; and (3) demographic characteristics of people who die by suicide, including gender (ie, female, male, or group suicidal event), age (ie, juvenile, youth, middle-aged, or older adults group), marital status (ie, unmarried, married, divorced, or widowed), and economic activity status (ie, student, economically active–employed, economically active–unemployed, or economically inactive) [43].

Second, we designed a social media evaluation framework based on the WHO guidelines to assess the quality of suicide reporting by mainstream publishers. We divided the content of the suicide reports into the categories harmful and helpful information [20,24,36]. The harmful information category included level of detail of the suicide description, disclosure of private information, and vividness of the reporting.

Despite their usefulness, the WHO guidelines were derived from press practices of traditional media, and the recommendations were not aligned with the features of social media. Hence, we adjusted several of the original items to improve the flexibility and applicability of the evaluation framework; for example, because social media posts do not have a layout order, avoid the prominent placement of suicide reporting was modified to avoid the inclusion of suggestive signs or emojis in suicide reporting. To improve the evaluation framework’s scientificity and operability, we removed the item about disclosure of private information about the person who died by suicide from harmful information after consulting with relevant experts because the meaning of *information* is too broad to operationalize. Finally, we achieved an evaluation framework containing 11 dichotomous items (ie, present=1, absent=0). The structure of the evaluation framework and item definitions and examples are shown in Table 1.

**Table 1:** Evaluation framework for the quality of social media suicide reporting.

Dimension, subdimension, and item				Example
**Harmful information**				
	**Level of detail of the suicide description**			
		Describes in detail the tools used for suicide or specific suicide actions, such as drug dosage.	“She took nearly 500 tablets of chlorpheniramine in her car.”	
		Provides specific information about the site of suicide event, such as the exact name of the bridge, street, community, and so on.	“Weikang Hotel, Nujiang Town, Tongjiang County, Sichuan Province.”	
	**Disclosure of private information**			
		Includes photographs or videos showing images of the person who died.	—^a^	
		Describes the content of the suicide note in detail.	“He expressed his apology to parents and left his bank card password in the suicide note.”	
		Includes interviews of immediate family members of the person who died by suicide.	“His father said in the interview that his daughter was often depressed after the accident.”	
	**Vividness of the reporting**			
		Attaches hashtags at the beginning or end of the post.	#The 13-year-old boy left home after being blamed by his father#	
		Uses suggestive characters or emoji to highlight the content.	“!!!,” “~~”	
		Includes the word *suicide* directly in the headline or discloses the cause of suicide.	“Confession failed, a man climbed to a high building to suicide.”	
**Helpful information**				
	Relates suicidal behaviors with mental disorders such as depression.		“This pregnant woman suffered from postpartum depression.”	
	Provides information on where to seek help such as a psychological consultation hotline or other public health service.		“Beijing Huilongguan Hospital psychological crisis intervention hotline: 800-810-1117.”	
	Provides professional knowledge about suicide prevention from psychologist experts or scholars.		“The psychiatrists said that [positive psychological intervention] can completely change the situation of mental illness.”	

^a^Not applicable.

We selected 3 well-trained graduate students who had majored in journalism and communication for coding; they used an explicit coding framework. We conducted a reliability test based on 100 sample data items, finding the following: the interrater reliability of suicide reporting characteristics ranged from 0.78 to 0.98 with an average of .91 and that of suicide reporting quality ranged from 0.73 to 1.0, with an average of 0.88. Based on prior research, both parts of the framework indicated strong interrater reliability [44]. To bridge understanding deviations regarding the coding and reach consensus throughout the research process, we held regular meetings with the coders.

## Results

### Analysis of Suicide Reporting

The number of suicide reports varied considerably among the analyzed media accounts (see Table 2). Overall, the Global Times showed the largest number of suicide reports (21.26%, 503/2366), whereas Guangming Daily showed the smallest number (0.89%, 21/2366), both being national publishers. In local publishers, the Shanghai Morning Posts showed the largest number (19.65%, 465/2366), second only to the national publisher Global Times. From the perspective of time, the number of suicide reports varied considerably between 2011 to 2019; the year 2018 showed the largest number of suicide reports (19.57%, 463/2366) and 2011, the lowest (1.61%. 38/2366).

**Table 2:** Suicide reporting by national and local Chinese publishers (n=2366).

Newspaper name	Suicide reports, n (%)
Global Times	503 (21.26)
Shanghai Morning Post	465 (19.65)
Chutian Metropolis Daily	420 (17.75)
Beijing News	330 (13.95)
Yangtze Evening Post	246 (10.40)
People’s Daily	121 (5.11)
Southern Metropolis Daily	92 (3.89)
China Daily	87 (3.68)
China Youth News	81 (3.42)
Guangming Daily	21 (0.89)

### Characteristics of Suicide Reporting on Social Media

Most reported suicide events occurred in urban areas (1454/1588, 91.56%; excluding 778 posts lacking information on site; see Table 3). Almost all suicide reporting disclosed the suicide method (2195/2366, 92.77%), with jumping off buildings (884/2195, 40.27%) and jumping into rivers (391/2195, 17.81%) being the most commonly reported. Despite being one of the most popular suicide methods in South and East Asia [45], reported events of drinking pesticide ranked fourth (172/2195, 7.84%).

Nearly 80% (1823/2366, 77.05%) of the reporting described the suicide attribution as stated in media reports, among these family conflict (446/1823, 24.47%), followed by emotional frustration (295/1823, 16.18%), financial difficulty (199/1823, 10.92%), and interpersonal conflicts (196/1823, 10.75%).

Regarding the demographic characteristics of people who died by suicide (Table 3), the number of females (1096/2326, 47.12%) was very close to males (1117/2326, 48.02%), and 4.86% (113/2326) reports were group suicidal events. More than 60% (1446/2366, 61.12%) of suicide reporting disclosed the person’s age, of which the youth group accounted for more than half (730/1446, 50.48%), followed by the juvenile (424/1446, 29.32%), middle-aged (174/1446, 12.03%), and older adults groups (118/1446, 8.16%). In addition, suicide reports on social media more frequently reported on suicidal behaviors of unmarried groups and students.

**Table 3:** Descriptive characteristics of suicide reporting on social media (n=2366).

Characteristics				Values, n (%)
**Suicide event characteristics**				
	**Event (n=2366)**			
		Suicide attempt	1308 (55.28)	
		Suicide death	1058 (44.72)	
	**Site (n=1588)**			
		Urban area	1454 (91.56)	
		Rural area	134 (8.44)	
	**Method (n=2195)**			
		Jumping off building	884 (40.27)	
		Jumping into river	391 (17.81)	
		Other way	212 (9.66)	
		Drink pesticide	172 (7.84)	
		Jumping off bridge	165 (7.52)	
		Hanging	119 (5.42)	
		Taking medicine	94 (4.28)	
		Wrist cutting	60 (2.73)	
		Charcoal burning	58 (2.64)	
		Multiples ways	40 (1.82)	
	**Attribution (factor) as stated in media reports (n=1823)**			
		Family conflict	446 (24.47)	
		Emotional frustration	295 (16.18)	
		Financial difficulty	199 (10.92)	
		Interpersonal conflict	196 (10.75)	
		Mental disorder	172 (9.43)	
		Study or work pressure	168 (9.22)	
		Other cause	157 (8.61)	
		Avoid responsibility	60 (3.29)	
		Physical disease	55 (3.02)	
		Loneliness or solitude	42 (2.30)	
		Multiple factors	33 (1.81)	
**Demographic characteristics**				
	**Gender (n=2326)**			
		Male	1117 (48.02)	
		Female	1096 (47.12)	
		Group suicidal event	113 (4.86)	
	**Age group (years; n=1446)**			
		Youth (19 to 35)	730 (50.48)	
		Juvenile (below 18)	424 (29.32)	
		Middle-age (36 to 65)	174 (12.03)	
		Older adults (over 65)	118 (8.16)	
	**Marital status (n=1292)**			
		Unmarried	927 (71.75)	
		Married	324 (25.08)	
		Divorced	31 (2.40)	
		Widowed	10 (0.77)	
	**Economic activity status (n=1062)**			
		Student	593 (55.84)	
		Economically active–employed	364 (34.27)	
		Economically inactive	60 (5.65)	
		Economically active–unemployed	45 (4.24)	

### Assessing Suicide Reporting Quality on Social Media Against WHO Guidelines

Our study demonstrated that harmful reporting practices were widespread on Chinese social media (Table 4); 41.50% (982/2366) of suicide reporting contained 3 or more harmful instances of information, while only 5.92% (140/2366) of the suicide reporting did not have any harmful information. Further, the vividness of the reporting subdimension had the highest mean (mean 0.35), followed by disclosure of private information (mean 0.27), and level of detail of the suicide description (mean 0.22).

Regarding vividness of the reporting, more than half of the suicide reporting either directly used the word “suicide” or disclosed the reasons for suicide in the headlines, practices that deviate considerably from the WHO guidelines to word headlines carefully. It is worth noting that the use of internet elements has become common on social media platforms; on the topic, this study showed that 37.19% (880/2366) of the suicide reporting used suggestive symbols (eg, multiple exclamation points or emojis). Furthermore, 13.99% (331/2366) of the suicide reporting attached hashtags at the beginning of the articles. These hashtags were highly visible because they were discussed by the public to a certain extent (eg, the hashtag #26-year-old female teacher fell off the building#).

Regarding the disclosure of private information, half (1172/2366, 49.54%) of the suicide reporting exposed images of the people who died, and nearly 30% (313/1172, 26.71%) were not pixelated. Additionally, 11.33% (268/2366) of suicide reporting disclosed the suicide note, with 41.8% (112/268) of the notes coming from the person’s social media platform (eg, on Weibo or WeChat). According to the WHO guidelines, social media posts and email information of people who die by suicide should not be disclosed, as these suicide notes may contain sensitive private information (eg, their debts or information on their interpersonal relationships).

Regarding level of detail of the suicide description, almost 24% (568/2366, 24.01%) of the suicide reporting described the suicide methods in detail (eg, “She closed the window, locked the door, and lit the charcoal fire”). Moreover, 20.08% (475/2366) of the suicide reporting provided specific information on the suicide site (eg, the exact names of streets, communities, or bridges).

Our results also indicated that Chinese mainstream publishers’ suicide reporting on social media has significant deficiencies regarding the provision of helpful information: only 1 of all 2366 suicide reports included all the helpful information outlined in evaluation framework for this study; in contrast, more than 85% (2017/2366, 85.25%) of the reporting did not provide any helpful information. WHO considers it important to emphasize the causal relationship between suicidal behavior and mental illness for suicide prevention efforts [20]. However, only 10.86% (257/2366) of the analyzed suicide reporting highlighted this relationship. Moreover, less than 6% (137/2366, 5.79%) of the suicide reporting provided suicide prevention knowledge for the public (eg, psychology experts’ advice on suicide prevention or official suicide statistics to highlight the importance of the suicide issue). Furthermore, only 2 reports provided direct information about support programs, which are essential for vulnerable groups seeking help; in contrast, the WHO guidelines suggest mass media publishers list available sources of support (eg, psychological intervention hotlines and community resources) for those in need [20].

**Table 4:** Assessing suicide reporting quality on social media against the WHO guidelines (n=2366).

Items			Value, n (%)
**Harmful information**			
	**Vividness of the reporting**		
		Headline contains suicide-related words	1292 (54.61)
		Suggestive symbols or emojis used	880 (37.91)
		Hashtag used	331 (13.99)
	**Disclosure of private information**		
		Exposure of images of people who died by suicide	1172(49.54)
		Interviews with relatives of people who died by suicide	376 (15.89)
		Disclosure of the suicide note	268 (11.33)
	**Level of detail of the suicide description**		
		Detailed description of the suicide method	568 (24.01)
		Detailed description of the suicide site	475 (20.08)
**Helpful information**			
	Emphasize relationship between suicide and mental disorders		257 (10.86)
	Provide suicide prevention knowledge		137 (5.79)
	Provide information about support programs		2 (0.08)

## Discussion

### Principal Findings

This study analyzed the characteristics of suicide reporting of the top 10 publisher accounts (in terms of number of followers) on Sina Weibo and assessed their suicide reporting quality against the WHO guidelines. Our analyses yielded the following findings: (1) Chinese mainstream publishers on social media most frequently reported on suicides that were stated to be associated with conflict and underreported suicides in older adults and rural residents, (2) these suicide reports provided widespread harmful information, especially concerning vividness of the reporting and disclosure of private information, and (3) these reports provided limited helpful information such as direct information about suicide support programs.

Analysis of suicide reporting characteristics revealed two features. First, the reporting of suicide events was found to be clickbait oriented, in which publishers tried their best to attract readers with a sensational and dramatic reporting style. Suicide is a complex phenomenon with multiple contributing factors. However, in this study, almost all suicide reports interpreted suicide as an unexpected event caused by a single factor, with interpersonal conflicts and conflicts leading to suicide (eg, by family conflict or emotional frustration) being the most commonly reported causes. Only 1.81% of posts published a multicausal explanation of suicides. This may be explained by the publisher intention to catch the reader’s eye and gain clicks by describing suicide news in a conflicted and dramatic manner [38].

Second, suicides of older adults and rural residents were underreported. Our study demonstrated that the analyzed social media accounts put a disproportionate amount of focus on young adult suicide while rarely reporting on suicides among older adults; in contrast, according to China’s official health data reports, the suicide rate of people aged over 65 years is the highest among all age groups. For example, the average suicide rate of urban residents aged 65 to 85 years in 2018 was about 5.5 times higher than that of people aged 20 to 40 years (13.10 vs 2.39 per 100,000) [46]. In addition, the suicide rate of rural residents in China has always been higher than that of urban residents [46]. The underreporting of older adult suicide found in this study is consistent with the findings of Fu et al [23] for traditional newspapers. This reporting bias may pose a considerable risk for suicide prevention efforts; for example, policymakers may acknowledge the lack of reports and promote an unbalanced distribution of health resources. This type of bias may lead to the neglect of the suicides of vulnerable people by the general population, thus hindering people’s ability to intervene and support those at risk for suicide. Given the specific context of the Chinese population, which has been experiencing rapid aging [47], such bias and the risk that it produces warrant great focus from researchers and practitioners.

In terms of suicide reporting quality, our results revealed that the analyzed social media accounts had low adherence to the WHO guidelines; this result echoed results found in previous Asian research [23-26]. Regarding harmful information, vividness of the reporting showed the worst performance; for traditional printed media, to reduce the risk of imitative suicide, publishers are advised to avoid placing suicide reporting in prominent positions of the printed news [20,38]. The research of Chu et al [25] found low compliance—23% for newspaper articles—with the guideline to avoid prominent placement like the front page, boxes, or similar, but 85% for internet-based media articles. Our findings are likely to explain these differences further. Namely, although there is no fixed layout on social media, our results indicated that it uses alternative ways to capture readers’ attention, such as using diverse internet elements (eg, hashtags and emojis). However, there are currently no standardized guidelines for their use. We are worried that this type of reporting style may be turning a serious public health issue into something that may be entertaining or even frivolous.

We also found that disclosure of private information showed a moderately problematic performance; almost half of the suicide reporting on the analyzed social media accounts exposed images of the person who died by suicide. This finding is concordant with the study of Fu et al [23] of Chinese newspapers (57.5%) [23]; one possible explanation is that, as prior studies showed, Chinese people may be generally insensitive to privacy issues [48,49]. This conclusion can also be supported by research conducted in India, where 21.5% of the reporting contained photos of the person who died by suicide [24]. Also, a study on Facebook found that only 24% of suicide news disclosed a photo or video with information about the suicide site [36].

Our findings demonstrated that level of detail of the suicide description showed relevant noncompliance ratios; specifically, 24.01% of the suicide reporting provided an explicit description of the suicide method. This result is roughly consistent with prior studies on print newspapers in China and India [23,24]; however, since the speed and breadth of social media is greater than that of traditional media, we believe that even if it is roughly an equal proportion, social media may have a more profound negative impact.

Regarding helpful information, we found that the analyzed Chinese social media accounts provided very limited information on suicide prevention. WHO has suggested that mass media endeavor eliminate the general public’s misconception about suicide and convey the view that mental illnesses and suicide are inseparable [20,50]. Unfortunately, less than 11% of the suicide reports in this study emphasized the relationship between mental health and suicidal behaviors. This result is consistent with previous research by Chu et al [25] which showed that less than 20% of the reported suicides (including in newspapers and websites) acknowledged the relationship between suicidal behavior and mental illness. However, in New Zealand, this proportion was more than 30% [51] and in Australia, it was more than 50% of the analyzed samples [15]. To some extent, this shows the different attribution patterns of suicidal behavior in Chinese and Western media. Regarding information on support programs, our results showed that only 2 reports attached direct information on support programs like psychological intervention hotlines. This result echoes the findings on traditional media, suggesting that both traditional and social media do an inefficient job of providing information about support programs [23-25]. In addition, a US study on suicide reporting on Facebook revealed that 16% of suicide news contained a hotline phone number, a higher percentage than in China but still low overall [36]. In summary, while social media has tremendous convenience, such as link support resources, the potential of mainstream publishers to provide helpful information on suicide is currently underused.

### Implications

For decades, academic and practical fields have been concerned with the quality of suicide reporting in the mainstream media. However, we have little information on how mainstream publishers report suicide via social media platforms. Although a recent study assessed suicide news on Facebook in English-speaking countries [36], empirical evidence on suicide reporting on Asian social media platforms, especially in China, is still very limited. Thus, we endeavored to examine adherence to WHO suicide guidelines on Chinese social media platforms; we intended to address the above mentioned gap by empirically investigating these reporting qualities.

Various studies have indicated that the degree of compliance with the WHO guidelines are affected by complex factors, including economic and media policy issues present in different countries [24,36,52]. Among them, culture is an important factor that cannot be ignored. This is evident in the Chinese and Western attributions regarding suicide. It is generally believed that China is an acquaintance society that attaches great importance to interpersonal relations. Therefore, Chinese society (including the media) focuses on suicide caused by interpersonal conflicts such as marriage and family conflicts. In contrast, the West tends to explain suicide in terms of pathology, emphasizing suicides that occur due to mental illness [53]. Our findings also confirm the cultural differences in suicide attribution between China and the West. Our study complements the current literature on suicide reporting in different cultural contexts, thereby indirectly supporting the cross-cultural study of suicide.

Our study has enormous practical implications for national—and potentially international—suicide prevention programs. First, for media practitioners, since we found that there was generally low compliance with WHO guidelines, we reiterate Chinese mainstream publishers’ role as gatekeepers on social media platforms by encouraging the strengthening of editorial review policies to limit harmful information and disseminate helpful information. Since media practitioners’ understanding and support of the guidelines will also affect the implementation of the guidelines [52], these publishers should focus on training and educating their social media professionals to enhance their knowledge of critical public health issues (eg, suicide).

Second, our study clarifies the role of the Ministry of Health in promoting responsible media practices; given that in China there are currently no national suicide reporting guidelines [54], one of the first tasks may be to develop national guidelines that align with the current condition of the media in the country. We suggest that an updated suicide reporting guideline document should fully consider the internet elements. Furthermore, considering that social media is constantly evolving and richer forms of media are likely to emerge in the future, the Ministry of Health should support the implementation of these guidelines and the development of supervisory tools to monitor and evaluate the quality of suicide reporting on social media on an ongoing basis.

Third, social organizations (eg, mental health institutions) can actively cooperate with mainstream publishers to increase dissemination of helpful information related to suicide on social media platforms. Prior studies have shown that stakeholders’ active participation in the development of guidelines effectively boosts compliance with the guidelines [52,55]. Therefore, while social organizations provide professional knowledge on suicide prevention, social media can ensure that this knowledge is highly disseminated.

Last, users can play an active role in suicide prevention. On an interactive information platform, every click made by social media users can be regarded as a second transmission. The promotion and discussion of suicide topics on social networks may increase negative thoughts of suicide by members of vulnerable groups. It is necessary to help users safely convey information about suicide through social media [56]. Our results suggest that users should refrain from retweeting suicide reports that contain harmful information, such as those containing the private information of the people who died by suicide, and instead spread suicide-related articles with rich educational materials (eg, on psychological interventions and suicide prevention-related knowledge).

### Limitations

This study has some limitations. First, since our data on suicide reporting came exclusively from the 10 most influential Chinese mainstream media accounts on Weibo, we did not include data from small-size media accounts, which may still play a meaningful role in particular and local issues. Thus, future evaluation research may include a greater variety of media types. In addition, our study only assessed the suicide reporting of the analyzed publishers on Weibo; thus, future research can extend the analysis to other Chinese social media platforms (eg, WeChat) and attempt to conduct cross-cultural research on the topic of suicide on English-language social media platforms.

### Conclusions

This study analyzed the characteristics of suicide reporting published by Chinese mainstream publishers via a social media platform and assessed the suicide reporting quality against WHO guidelines. Our findings illustrated that suicide reporting on social media by mainstream publishers in China disseminated too much harmful information, especially concerning the vividness of the reporting and disclosure of private information. Conversely, they provided minimal helpful information. Considering the tremendous information dissemination power of social media platforms, we highlight the need for the development of national suicide reporting guidelines that apply to the new media environment and local cultural backgrounds. We also recommend that social media practitioners, health care institutions, social organizations, and the public work together to promote responsible suicide reporting in the social media environment.

## Abbreviations

WHO: World Health Organization
